# Evaluating audio computer assisted self-interviews in urban south African communities: evidence for good suitability and reduced social desirability bias of a cross-sectional survey on sexual behaviour

**DOI:** 10.1186/1471-2288-13-11

**Published:** 2013-01-31

**Authors:** Roxanne Beauclair, Fei Meng, Nele Deprez, Marleen Temmerman, Alex Welte, Niel Hens, Wim Delva

**Affiliations:** 1 The South African Department of Science and Technology/National Research Foundation (DST/NRF) Centre of Excellence in Epidemiological Modelling and Analysis (SACEMA), Stellenbosch University, 19 Jonkershoek Road, Stellenbosch, 7600, South Africa; 2 International Centre for Reproductive Health, Ghent University, De Pintelaan 185, Ghent, 9000, Belgium; 3 Center for Statistics, Interuniversity Institute of Biostatistics and Statistical Bioinformatics, Hasselt University, Diepenbeek, Belgium; 4 Faculty of Medicine and Health Sciences, Ghent University, De Pintelaan 185, Ghent, 9000, Belgium; 5 Centre for Health Economic Research and Modeling Infectious Diseases, Vaccine and Infectious Disease Institute, University of Antwerp, Wilrijk, Belgium

**Keywords:** ACASI, Sexual behaviour, Social desirability bias, Self-reported data, Gender, South Africa

## Abstract

**Background:**

Efficient HIV prevention requires accurate identification of individuals with risky sexual behaviour. However, self-reported data from sexual behaviour surveys are prone to social desirability bias (SDB). Audio Computer-Assisted Self-Interviewing (ACASI) has been suggested as an alternative to face-to-face interviewing (FTFI), because it may promote interview privacy and reduce SDB. However, little is known about the suitability and accuracy of ACASI in urban communities with high HIV prevalence in South Africa. To test this, we conducted a sexual behaviour survey in Cape Town, South Africa, using ACASI methods.

**Methods:**

Participants (n = 878) answered questions about their sexual relationships on a touch screen computer in a private mobile office. We included questions at the end of the ACASI survey that were used to assess participants’ perceived ease of use, privacy, and truthfulness. Univariate logistic regression models, supported by multivariate models, were applied to identify groups of people who had adverse interviewing experiences. Further, we constructed male–female ratios of self-reported sexual behaviours as indicators of SDB. We used these indicators to compare SDB in our survey and in recent FTFI-based Demographic and Health Surveys (DHSs) from Lesotho, Swaziland, and Zimbabwe.

**Results:**

Most participants found our methods easy to use (85.9%), perceived privacy (96.3%) and preferred ACASI to other modes of inquiry (82.5%) when reporting on sexual behaviours. Unemployed participants and those in the 40–70 year old age group were the least likely to find our methods easy to use (OR 0.69; 95% CI: 0.47–1.01 and OR 0.37; 95% CI: 0.23–0.58, respectively). In our survey, the male–female ratio for reporting >2 sexual partners in the past year, a concurrent relationship in the past year, and > 2 sexual partners in a lifetime was 3.4, 2.6, and 1.2, respectively— far lower than the ratios observed in the Demographic and Health Surveys.

**Conclusions:**

Our analysis suggests that most participants in our survey found the ACASI modality to be acceptable, private, and user-friendly. Moreover, our results indicate lower SDB than in FTFI techniques. Targeting older and unemployed participants for ACASI training prior to taking the survey may help to improve their perception of ease and privacy.

## Background

Sexual behavioural research largely depends on self-reported data in the absence of more objective biomarkers. Ensuring the validity of these data is a common challenge faced by traditional survey methods. Specifically, self-reports of sensitive issues such as engagement in multiple, concurrent sexual relationships may be subject to social desirability bias (SDB)— or the inclination to report behaviours that are viewed in a favourable way by society. For sexual behaviours the bias is often gendered because of sexual double standards in society, which frequently lead men to over-report and women to under-report certain behaviours [1-4].

For several countries in Africa, young unmarried women tend to report a later age for their first sexual experience compared to young unmarried men [5]. In regard to sexual concurrency —overlapping sexual relationships where a new sexual relationship starts before an existing relationship ends— several studies from Africa indicate that men are far more likely to report having multiple sexual partners at once, or extra-marital partners, compared to women [6-9]. Women have also been found to report fewer non-spousal partners in the past year compared to men [10]. Gendered discrepancies also exist in reporting of condom use in Africa. Men consistently are more likely to report having ever used condoms [6] and always using condoms with all of their partners [9,11] than women. A study conducted specifically to test SDB among married couples, found that a higher percentage of husbands reported that they used condoms with their spouse, than their wives did [12].

When participants consciously modify their answers to project a socially ‘appropriate’ response, this is called impression management [13], and the effects of it can theoretically be ameliorated by changing the design of the study. To combat the problem of SDB, several studies have investigated how mode of inquiry might reduce this phenomenon. Audio Computer-Assisted Self-Interviewing (ACASI) techniques have emerged as a way to curtail SDB because they allow participants to answer questions in privacy by using a computer to read the question, while simultaneously listening to an audio recording of the questions being asked. Several studies have attempted to evaluate the accuracy of ACASI-generated survey results and have produced disparate results. For example, studies done in India have shown that women reported forcibly being touched [14] and domestic violence less often in ACASI compared to traditional Face-to-face-interviewing (FTFI) methods [15]. In the U.S. a study was conducted to see if reported condom use in an ACASI could be substantiated by a Y-chromosome polymerase chain reaction (Yc-PCR) test to detect sperm in vaginal fluid. It demonstrated that even with ACASI methods, there were still inconsistencies between the Yc-PCR and reported results [16]. However, in the African context ACASI methods seemed to precipitate the reporting of more sexual behaviours [17]. Increases in the reporting of number of sexual partners [18], two or more sex partners at once [19], and for women, having had sex with a relative, stranger, or older man [20] in ACASI compared to FTFI seem to indicate that participants in Africa may be more comfortable answering questions about sexual behaviour when they have access to greater privacy.

These discrepant findings about ACASI compared to FTFI are indicative of the need for more context-specific research on ACASI methods. Particularly, since little is known about the validity of ACASI and its suitability for the context of urban, disadvantaged communities in South Africa, where the burden of HIV/AIDS is among the highest in the world. We conducted a cross-sectional survey in three urban communities of Cape Town, South Africa that are known to have a high prevalence of HIV. As a part of the survey, we asked participants questions about their interview experience. In this paper, we report the results of these questions and we compare key summary statistics known to be highly sensitive to SDB from our survey with those from the Demographic and Health Surveys (DHS), conducted in Lesotho, Swaziland, and Zimbabwe [21-23].

## Methods

### The Cape Town sexual behaviour ACASI survey

We administered a cross-sectional sexual behaviour survey (n = 878) in three urban, economically disadvantaged communities around Cape Town from June 2011 to February 2012. The survey took a sub-sample of participants from the Zambia South Africa TB and AIDS Reduction Study (ZAMSTAR), a community randomised trial that aimed to reduce the prevalence of TB and HIV using novel public health interventions. The primary objective of the survey analyzed here was to look at associations between HIV status, sexual connectedness and age-disparate relationships. To this end, we explored participants’ one-year sexual histories focusing on dates of sexual activity episodes, coital frequency, condom use, age differences between partners, as well as drug and alcohol use at the time of first sexual intercourse. The complete study design and protocol are explained in further detail elsewhere [24].

The questionnaire was conducted in a safe, mobile office space using ACASI on touch screen computers. The ACASI software was developed by an independent software engineering company. Participants listened to audio recordings of questions and response options through headphones. They were allowed to take the survey in their choice of language: English, Afrikaans, or isiXhosa. Furthermore, the questionnaire was set up along a temporal trajectory with the onset, dissolution, and duration of each relationship episode displayed on a touch screen timeline for the participant to see, using different colours for each partner. Participants had the option of listening to the questions over again, or skipping the question if they did not want to answer it. At the end of the survey we added four questions to assess the participant’s experience with the ACASI mode of inquiry. It has been established that any survey method needs to be user-friendly and offer privacy, if participants are to answer truthfully and give full attention to the questions for the entire duration of the questionnaire [13]. Therefore, we surmised that these four questions would give the best indication of whether or not participants had a positive experience with a survey modality that was new to their community.

Of 1857 people randomly sampled from the ZAMSTAR sampling frame, we were able to locate the addresses of 1115 (60.0% contact rate). For 197 people, the reason for non-contact after three attempts was unknown, while for respectively 511 and 34, relocation to an unknown new address and death was documented. An additional 87 candidate participants were excluded, primarily due to visual or physical impairments that rendered participation in the study impossible. Of the remaining 1028 who were eligible, 878 (85.4% response rate) took the survey [25].

For this analysis, we excluded participants that had missing ages (n = 7), were younger than 15 years old (n = 1), and were older than 70 years old (n = 26). We also excluded participants that did not identify as a man or woman (n = 30). Additionally, we excluded participants (n = 16) that did not identify as a black or coloured person, as these racial groups are not typical of populations that have a high prevalence of HIV in South African urban communities. The term *coloured* refers to racially mixed descendants of Europeans, indigenous populations and slaves from South and East Asia.

### Statistical analysis

Our analysis is divided into three parts. First we computed descriptive statistics for the four questions that assessed the participants’ interview experience. We asked about the participant’s ease of use (very easy/somewhat easy/somewhat difficult/very difficult/I don’t know), perceived confidentiality (very private/somewhat private/no privacy/I don’t know) and self-reported truthfulness of their answers to the survey questions (all questions answered truthfully/most truthfully/some truthfully, some dishonestly/most dishonestly/all dishonestly/I don’t know). Finally, we asked them to indicate their preferred medium of answering questions about sexual behaviour (touch screen computer with ACASI/researcher-administered verbal questionnaire/self-administered written questionnaire/telephonic survey/I don’t know).

Second, we conducted logistic regression analyses to identify groups of people that were less likely to have a positive experience with the interview. Five different univariate models were constructed for each of the following outcome variables: ease of use (easy/difficult), perceived confidentiality (private/no privacy), truthfulness (all truthfully/some dishonesty), and mode of inquiry (ACASI/other type). The univariate models examined the following variables as predictors of each of the outcomes: race (black/coloured), age (15–24/25–39/40–70), gender (male/female), education level (primary/secondary/tertiary), and employment status (employed/unemployed). Next, we constructed multivariate logistic regression models, adjusting for all of the predictors. However, here we present the results of the univariate analysis because we were primarily concerned with finding predictors of adverse experiences using marginal associations. Additionally, we thought that unadjusted ORs would be easier for others to interpret for their own settings if they are looking for ‘at-risk’ individuals to target for training in their own ACASI survey.

Finally, we attempted to assess the extent to which our ACASI survey was successful in reducing SDB. While its impossible to ascertain how accurate our results were, since the truth is unknown, we can attempt relative assessments of accuracy by comparing responses, by gender, among results from surveys that use different modes of inquiry. To this end, we constructed male–female prevalence ratios of self-reported sexual behaviours, as indicators of SDB, and we used these indicators to compare SDB in our survey and in recent FTFI-based, nationally representative DHSs from Lesotho, Swaziland, and Zimbabwe [21-23]. These three countries were chosen as comparison cases based on the assumptions that their cultural and behavioural context was most similar to South Africa. Specifically, we compared the male–female ratios for the variables: *> 2 partners in the past year*, *> 2 total lifetime number of sexual partners*, and *had a concurrent relationship in the past year*. Corresponding with the DHS data analysis, described in detail elsewhere [26], we defined concurrency as having two or more partners that overlapped in time in the year preceding the survey. The ratios were constructed by dividing the prevalence of the behavior reported for men, by the prevalence of the behavior reported for women. For this section of the analysis we limited our study population to the same categories of participants included in the DHS working paper on concurrent sexual partnerships in order to make our results more directly comparable with theirs. Thus, we made additional exclusions of participants who were coloured (n = 196) and older than 49 years old (n = 99).

For each of these variables, we assumed that men tend to over-report sexual behaviours, and women tend to under-reported them. If the prevalence’s of behaviours for men and women were in truth not as divergent as reported by FTFI-based surveys, we would expect these surveys to overestimate the true male–female ratios. Therefore, if our survey, that used ACASI methods, produces smaller male–female ratios for these behaviours, compared to the DHS surveys, we could expect our estimate to be closer to the truth, since male over-reporting and female under-reporting is minimized. We will calculate 95% confidence intervals for all ratios using Fisher’s exact methods of estimation. All data analyses were performed using R [27].

### Ethical approval

The study was approved by the Stellenbosch University Health Research Ethics Committee (N11/03/093). Written, informed consent was obtained for each respondent prior to administration of the questionnaire.

## Results

After exclusions, we were able to evaluate the ACASI user experience for 798 participants. Our study population was comprised mostly of women (68%) and participants identifying as black (75%). The median age of participants was 35.5 years old (IQR 26–46). Most participants chose to take the survey in isiXhosa language (66.5%), followed by Afrikaans (21.9%) and English (11.4%). Table 1 displays the results from our questions about interview modality. A majority of participants found that using ACASI was easy (‘very easy’: 67.2% and ‘somewhat easy’: 18.7%). Most participants also found that the ACASI method offered a certain level of privacy (‘very private’: 88.7% and ‘somewhat private’: 7.6%). Eighty-six percent of the participants claimed to have answered all questions truthfully. When questioned about their preferred mode of inquiry 82.5% said they would like to do similar surveys about sexual behaviour using a touch screen computer with ACASI.

**Table 1:** Participant experience with ACASI mode of inquiry in cross-sectional sexual behaviour survey

	**n**	**%**
**N**	798	
**Ease**		
**Very easy**	536	67.2
**Somewhat easy**	149	18.7
**Somewhat difficult**	84	10.5
**Very difficult**	28	3.5
**I don’t know**	1	0.1
**Confidentiality**		
**Very private**	708	88.7
**Somewhat private**	61	7.6
**No privacy**	26	3.3
**I don’t know**	3	0.4
**Truthfulness**		
**Answered all truthfully**	683	85.6
**Answered most truthfully**	84	10.5
**Answered some truthfully, some dishonestly**	13	1.6
**Answered most dishonestly**	9	1.1
**Answered all dishonestly**	7	0.9
**I don’t know**	2	0.3
**Preferred mode of inquiry**		
**Touch screen computer with ACASI**	658	82.5
**Researcher-administered verbal questionnaire**	66	8.3
**Self-administered written questionnaire**	38	4.8
**Telephonic survey**	36	4.5
**I don’t know**	0	0.0

Table 2 shows the results of the univariate models, developed to identify the groups of participants most likely to have a positive/negative user experience. Female participants (OR 1.68; 95% CI: 1.23–2.29) and those with a secondary (OR 2.00; 95% CI: 1.48–2.71) or tertiary education (OR 7.06; 95% CI: 1.62–30.82) were more likely to find the ACASI questionnaire easy to use, while those who were in the oldest age group (OR 0.37; 95% CI: 0.23–0.58 and the unemployed (OR 0.69; 95% CI: 0.47–1.01) were less likely to find it easy. Participants aged 25–39 (OR 2.10; 95% CI: 1.16–3.81) were more likely to view their experience as being confidential. Coloured participants were less likely to think our methods granted privacy (OR 0.46; 95% CI: 0.29–0.74). The oldest age group (OR 1.81; 95% CI: 1.06–3.09) and those with a secondary education (OR 1.53; 95% CI: 1.02–2.30) were the groups most likely to answer all questions truthfully. Participants with a secondary education (OR 2.40; 95% CI: 1.64–3.50) were the most likely to choose touch screen ACASI survey as their preferred mode of inquiry, while those who belonged to the oldest age group (OR 0.44; 95% CI: 0.24–0.79) were less likely to choose this modality.

**Table 2:** Unadjusted odds ratios (ORs) for predictors of adverse interview experiences

	**Ease OR (95% CI)**	**Confidentiality OR (95% CI)**	**Truthfulness OR (95% CI)**	**Mode of inquiry OR (95% CI)**
n	791	789	790	792
Age				
15–24 years	1.00	1.00	1.00	1.00
25–39 years	0.71 (0.45–1.14)	2.1 (1.16–3.81)	1.42 (0.85–2.37)	0.58 (0.32–1.07)
40–70 years	0.37 (0.23–0.58)	1.48 (0.84–2.60)	1.81 (1.06–3.09)	0.44 (0.24–0.79)
Gender				
Male	1.00	1.00	1.00	1.00
Female	1.68 (1.23–2.29)	1.40 (0.88–2.21)	1.02 (0.67–1.56)	1.03 (0.70–1.52)
Race				
Black	1.00	1.00	1.00	1.00
Coloured	1.03 (0.73–1.45)	0.46 (0.29–0.74)	0.88 (0.56–1.39)	1.34 (0.86–2.11)
Education				
None or primary	1.00	1.00	1.00	1.00
Secondary	2.00 (1.48–2.71)	1.39 (0.88–2.18)	1.53 (1.02–2.30)	2.40 (1.64–3.50)
Tertiary	7.06 (1.62–30.82)	0.89 (0.25–3.14)	0.89 (0.29–2.75)	2.00 (0.57–6.97)
Employment Status				
Employed	1.00	1.00	1.00	1.00
Unemployed	0.69 (0.47–1.01)	0.87 (0.50–1.52)	0.92 (0.56–1.51)	0.88 (0.55–1.39)

*OR*, Unadjusted Odds Ratio.

*CI*, Confidence Interval.

Table 3 provides a comparison of different reported sexual behaviours, by men and women, in our survey with three different FTFI-based surveys in southern Africa. In this comparison, we used only 503 of our original participants, creating a subset of participants with demographics most closely resembling the DHS sample population. For all sexual behaviours examined, our survey produces smaller male–female prevalence ratios than the DHSs in Lesotho, Swaziland, and Zimbabwe. The ratio of men to women reporting greater than 2 partners in the past year in our survey was 3.5 (95% CI: 2.3–5.2) compared to 10.6 (95% CI: 7.3–15.2) in Lesotho, 20.0 (95% CI: 7.1–72.9) in Swaziland, and 22.0 (95% CI: 9.4–49.0) in Zimbabwe. For the prevalence of greater than 2 lifetime partners, our survey produced a ratio of 1.2 (95% CI: 1.0–1.3) compared to 2.1 (95% CI: 2.0–2.2) in Swaziland and 4.9 (95% CI: 4.6–5.2) in Zimbabwe. Our survey also produced a ratio of 2.6 (95% CI: 1.9–3.6) for respondents reporting a concurrent relationship in the past year (Swaziland 15.4, 95% CI: 10.7–21.7; Zimbabwe 11.3, 95% CI: 8.8–16.0). Finally, Table 3 also shows that the prevalence’s of all reported behaviours are larger for men and women in our survey compared to the others.

**Table 3:** Percent of respondents reporting risk behaviours by gender in three DHS surveys using face-to-face interviews and the ACASI administered Cape Town Sexual Behaviour Survey

**Survey**	**Interview mode**	**Sexual behavior characteristics**								
		**> 2 Partners in past year**			**> 2 Lifetime partners**			**Had a concurrent partnership**		
		**Male (%)**	**Female (%)**	**PR**	**Male (%)**	**Female (%)**	**PR**	**Male (%)**	**Female (%)**	**PR**
**Cape Town Sexual Behavior Survey**	ACASI	35.8	10.4	3.4	71.4	61.6	1.2	41.5	15.7	2.6
**Lesotho DHS**	FTFI	7.4	0.7	10.6	63.1	NA^*^	NA^*^	17.7	NA^*^	NA^*^
**Swaziland DHS**	FTFI	2.0	0.1	20.0	67.9	32.8	2.1	12.3	0.8	15.4
**Zimbabwe DHS**	FTFI	2.2	0.1	22.0	60.9	12.5	4.9	7.9	0.7	11.3

*PR*, male–female prevalence ratio.

*ACASI*, Audio Computer-Assited Self-Interviewing.

*FTFI*, Face-to-Face Interviewing.

^*^ The DHS did not report prevalences for these female behaviors and therefore, the PR could not be calculated.

## Discussion

We assessed the user-friendliness, privacy and truthfulness of an ACASI-based sexual behaviour survey that was administered in disadvantaged, urban communities in Cape Town, South Africa. We found that an overwhelming majority of our participants preferred ACASI on touch screen computers to other modes of inquiry and that they answered all questions truthfully, probably owing in large part to participants’ perceived ease of use and privacy.

Despite the predominately positive experience, our analysis indicates that more effort may be required to improve the user experience for certain subgroups of the South African population. For instance, respondents over the age of 40 were less likely to think ACASI was easy to use, which might explain why they were also less likely to prefer ACASI as a mode of inquiry. Indeed, others have found that older generations are often intimidated by computers [28]. These results are not surprising given that computer literacy skills are only beginning to be taught in disadvantaged communities in South Africa. Moreover, unemployed participants found ACASI methods difficult to use probably due to the lack of computer exposure from a work environment. Since these groups of people found the survey challenging, it may have affected their recall of information due to the additional cognitive strain of navigating ACASI [13].

We also found that coloured participants were less likely to find our methods private. We suspect the reason for this is that a plurality of the coloured participants in our survey belonged to the oldest age group. McCallum and Peterson postulate that younger generations may find it more socially acceptable to share sensitive information due to changing views of privacy in the new technology age [13]. In fact, among Cape Town youth, there is a high use of mobile internet for instant messaging, digital media, and social networking [29], making their standards for privacy different from older generations. It should also be noted that in the multivariate analysis, adjusting for all of the potential predictors, the predictors did not change, only the size of the effects.

Our results also indicate that in this context, ACASI may produce more accurate results by partially removing some of the gendered SDB. In our survey the gender gap between reported sexual behaviours for men and women was narrowed, compared to the DHS that used FTFI techniques. Additionally, all of the sexual behaviours were reported more frequently in our survey than in the DHSs. This is particularly noticeable when looking at female reports of *> 2 partners in the past year* and *had a concurrent relationship*: less than 1% of women reported these behaviors in all DHS studies. This could be due to many different confounders or cultural differences between the two populations. However, we believe such a large difference in reported behaviours is most likely due to increased perception of privacy, which made our participants feel more at ease when answering intimate questions [30].

Our study had some limitations, which may affect the interpretation of our results. Firstly, we recognise that the questions we asked with respect to user experience, may also be subject to forms of bias. However, we believe that the bias would be minimal because typical forms of survey bias (e.g. inaccurate recall, social desirability, etc.) were probably not factors in answering these specific questions. Secondly, the positive results from the first two parts of our analysis may have been confounded by additional mechanisms we built into the survey to reduce bias, other than the interview mode. For one, we made use of a visual timeline to help respondents recall beginning and end dates of distinct episodes within each of their relationships. These visual cues have been demonstrated to encourage internal consistency in reporting relationship histories [31,32]. Additionally, our survey was relatively short, asking only a few questions for each reported relationship episode and partner, thus minimizing fatigue bias compared to other long surveys, such as DHSs. While these are an overall strength of the survey, they may have contributed to the positive user experience, as much or more than the ACASI itself.

The greatest strength of this study is that it is one of few sexual behaviour surveys that measured the user experience of the instrument [33]. This allowed us not only to find a proxy for how accurate our results were, but it enabled us to quantitatively assess how appropriate our methods were for the communities where the survey was administered. By asking questions about user experience we were able to determine that old age groups and unemployed people in similar contexts may be at risk for having an adverse experience with ACASI and consequently inaccurately reporting their results. Going forward, this knowledge will allow researchers to target these at-risk groups and provide more assistance and training for them before conducting a survey with similar methods.

## Conclusions

The results of our study indicate that ACASI methods are not only suitable for disadvantaged, urban African contexts, but they may considerably reduce SDB in sexual behaviour surveys. By targeting groups at-risk for finding ACASI methods challenging, and providing them with additional support and training prior to taking the survey, the accuracy of data may be improved. However, future work needs to be done on how to best train these people to make them feel comfortable with computerized survey methods. Correspondingly, more work needs to be done on how computer literacy and numeracy training affect the validity of ACASI responses.

## Competing interests

The authors of this manuscript have no competing interests.

## Authors’ contributions

RB, ND, MT, AW, NH and WD jointly designed the ACASI survey. RB coordinated data collection and wrote the first draft of the manuscript. FM conducted the statistical analysis. NH supervised the data analysis. All authors contributed during the editing process and approved the final, submitted manuscript.

## Pre-publication history

The pre-publication history for this paper can be accessed here:

http://www.biomedcentral.com/1471-2288/13/11/prepub

## References

[B1] Schroder KE Carey MP Vanable PA Methodological challenges in research on sexual risk behavior: I. Item content, scaling, and data analytical options Ann Behav Med 2003 26 2 76 103 10.1207/S15324796ABM2602_02 14534027 PMC2452993

[B2] Crawford M Popp D Sexual double standards: a review and methodological critique of two decades of research J Sex Res 2003 40 1 13 26 10.1080/00224490309552163 12806528

[B3] Boily MC Alary M Baggaley RF Neglected issues and hypotheses regarding the impact of sexual concurrency on HIV and sexually transmitted infections AIDS Behav 2012 16 2 304 311 10.1007/s10461-011-9887-0 21279678

[B4] Wellings K Collumbien M Slaymaker E Singh S Hodges Z Patel D Bajos N Sexual behaviour in context: a global perspective Lancet 2006 368 9548 1706 1728 10.1016/S0140-6736(06)69479-8 17098090

[B5] Zaba B Pisani E Slaymaker E Ties Boerma J Age at first sex: understanding recent trends in African demographic surveys Sex Transm Infect 2004 80 Suppl II ii28 ii35 15572637 10.1136/sti.2004.012674 PMC1765851

[B6] Clark S Kabiru C Zulu E Do Men and women report their sexual partnerships differently? evidence from kisumu, kenya Int Perspect Sex R H 2011 37 4 181 190 10.1363/3718111 PMC3383815 22227625

[B7] Mnyika KS Klepp KI Kvale G OleKingori N Determinants of high-risk sexual behaviour and condom use among adults in the Arusha region, Tanzania Int J STD AIDS 1997 8 3 176 183 10.1258/0956462971919840 9089028

[B8] Nnko S Boerma JT Urassa M Mwaluko G Zaba B Secretive females or swaggering males? An assessment of the quality of sexual partnership reporting in rural Tanzania Soc Sci Med 2004 59 2 299 310 10.1016/j.socscimed.2003.10.031 15110421

[B9] Prata N Vahidnia F Fraser A Gender and relationship differences in condom use among 15-24-year-olds in Angola Int Fam Plan Perspec 2005 31 4 192 199 10.1363/3119205 16439347

[B10] Buve A Lagarde E Carael M Rutenberg BJRF Glynn JR Laourou MEA Chege J Sukwa T Interpreting sexual behaviour data: validity issues in the multicentre study on factors determining the differential spread of HIV in four African cities AIDS 2001 15 suppl 4 S117 S126 11686460 10.1097/00002030-200108004-00013

[B11] Meekers D Klein M Determinants of condom use among young people in urban Cameroon Stud Family Plann 2002 33 4 335 346 10.1111/j.1728-4465.2002.00335.x 12553189

[B12] Cordero-Coma J Breen R HIV prevention and social desirability: husband-wife discrepencies in reports of condom Use J Marriage Fam 2013 74 601 613

[B13] McCallum EB Peterson ZD Investigating the impact of inquiry mode on self-reported sexual behavior: theoretical considerations and review of the literature J Sex Res 2012 49 2–3 212 226 22380589 10.1080/00224499.2012.658923

[B14] Jaya Hindin MJ Ahmed S Differences in young people’s reports of sexual behaviors according to interview methodology: a randomized trial in India Am J Public Health 2008 98 1 169 174 10.2105/AJPH.2006.099937 18160677 PMC2156054

[B15] Rathod SD Minnis AM Subbiah K Krishnan S ACASI and face-to-face interviews yield inconsistent estimates of domestic violence among women in India: The Samata Health Study 2005–2009 J Interpers Violence 2011 26 12 2437 2456 10.1177/0886260510385125 21282116 PMC3126890

[B16] Rose E Diclemente RJ Wingood GM Sales JM Latham TP Crosby RA Zenilman J Melendez J Hardin J The validity of teens’ and young adults’ self-reported condom use Arch Pediatr Adolesc Med 2009 163 1 61 64 10.1001/archpediatrics.2008.509 19124705

[B17] Dolezal C Marhefka SL Santamaria EK Leu CS Brackis-Cott E Mellins CA A comparison of audio computer-assisted self-interviews to face-to-face interviews of sexual behavior among perinatally HIV-exposed youth Arch Sex Behav 2012 41 2 401 410 10.1007/s10508-011-9769-6 21604065 PMC3621976

[B18] Phillips AE Gomez GB Boily MC Garnett GP A systematic review and meta-analysis of quantitative interviewing tools to investigate self-reported HIV and STI associated behaviours in low- and middle-income countries Int J Epidemiol 2010 39 6 1541 1555 10.1093/ije/dyq114 20630991

[B19] Kissinger P Rice J Farley T Trim S Jewitt K Margavio V Martin DH Application of computer-assisted interviews to sexual behavior research Am J Epidemiol 1999 149 10 950 954 10.1093/oxfordjournals.aje.a009739 10342804

[B20] Hewett P Mensch B Erulkar A Consistency in the reporting of sexual behavior by adolescent girls in Kenya: a comparison of interviewing methods Sex Transm Infect 2004 80 Suppl 2 ii43 ii48 15572639 10.1136/sti.2004.013250 PMC1765856

[B21] Central Statistical Office (CSO) S, Inc MI Swaziland Demographic and Health Survey:2006–07 Demographic and Health Survey 2008 Mbabane, Swaziland: Central Statistical Office Macro International Inc

[B22] Central Statistical Office (CSO) Z, Inc MI Zimbabwe Demographic and Health Survey 2005–06 2007 Calverton, Maryland: CSO and Macro International Inc

[B23] Macro O Ministry of Health and Social Welfare (MOHSW) L, (BOS) Bos Lesotho Demographic and Health Survey 2004 2005 Calverton, Maryland: MOH, BOS, and ORC Macro

[B24] Delva W Beauclair R Welte A Vansteelandt S Hens N Aerts M du Toit E Beyers N Temmerman M Age-disparity, sexual connectedness and HIV infection in disadvantaged communities around Cape Town, South Africa: a study protocol BMC Public Health 2011 11 616 10.1186/1471-2458-11-616 21810237 PMC3161892

[B25] The American Association for Public Opinion Research Standard Definitions: Final Dispositions of Case Codes and Outcome Rates for Surveys 2011 7 AAPOR

[B26] Mishra V Assche B-V Concurrent Sexual Partnerships and HIV Infection: Evidence from National Population-Based Surveys 2009 Calverton, Maryland: Macro International Inc, DHS Working Papers No 62

[B27] R Development Core Team: R A language and environment for statistical computing 2005 Vienna: R Foundation for Statistical Computing

[B28] Feigelson ME Dwight SA Can asking questions by computer improve the candidness of responding? A meta-analytic perspective Consulting Psychol J: Pract Res 2000 52 4 248 255

[B29] Kreutzer T Generation Mobile: Online and Digital Media Usage on Mobile Phones among Low-Income Urban Youth in South Africa 2009 Cape Town: University of Cape Town

[B30] Lanhaug LF Sherr L Cowan FM How to improve the validity of sexual behavior reporting: systematic review of questionnaire delivery modes in developing countries Trop Med Int Health 2010 15 3 362 381 10.1111/j.1365-3156.2009.02464.x 20409291 PMC3321435

[B31] Kabiru CW Luke N Izugbara CO Zulu E The Correlates of HIV Testing and Impacts on Sexual Behaviour: evidence from a life history study of young people in Kisumu, Kenya BMC Public Health 2010 10 412 10.1186/1471-2458-10-412 20624323 PMC2912815

[B32] Luke N Clark S Zulu E The Relationship History Calendar: Improving Sexual Behaviour Data among Youth in Developing Country Settings 2008 New Orleans, USA: Population Association of America (PAA) Conference

[B33] Estes L Lloyd L Teti M Raja S Bowleg L Perceptions of Audio Computer-Assisted Self-Interviewing (ACASI) among Women in an HIV-Positive Prevention Program PLoS One 2010 5 2 e9149 10.1371/journal.pone.0009149 20161771 PMC2818842

